# Supplementary material for: Investigation of The Cellular Response to Bone Fractures: Evidence for Flexoelectricity
Source: Sci Rep. 2020 Jan 14;10:254. doi: 10.1038/s41598-019-57121-3 (PMC6959267; doi:10.1038/s41598-019-57121-3)
Supplement: Supplementary file 1 — Supplementary Information. [file 41598_2019_57121_MOESM1_ESM.pdf]

SUPPLEMENTARY INFORMATION

EFFECT OF FRACTURE FLEXOELECTRICITY ON BONE CELLS

Raquel Núñez-Toldrà [a,b,¥,*], Fabian Vasquez-Sancho [a,c,¥], Nathalie Barroca [a], Gustau Catalan [a,d,*]

[a] Institut Catala de Nanociencia i Nanotecnologia (ICN2), CSIC and the Barcelona Institute of Nanoscience and Nanotecnology (BIST), Bellaterra 08193, Barcelona.

[b] National Heart & Lung Institute, Imperial College London, London W12 0NN, UK

[c] Centro de Investigación en Ciencia e Ingeniería de Materiales, Universidad de Costa Rica, San José 11501, Costa Rica

[d] Institució Catalana de Recerca i Estudis Avançats (ICREA), Barcelona.

[¥] These authors contributed equally

***Corresponding authors at:**
Institut Catala de Nanociencia i Nanotecnologia (ICN2), CSIC and the Barcelona Institute of Nanoscience and Nanotecnology (BIST). Bellaterra 08193, Barcelona

E-mail: r.nunez-toldra@imperial.ac.uk (Raquel Nuñez-Toldra); gustau.catalan@icn2.cat (Gustau Catalan)

Diffraction pattern of Hydroxyapatite

The diffraction pattern of hydroxyapatite was measured to discard other impurity phases that could appear during the thermal treatment to make the pellet.

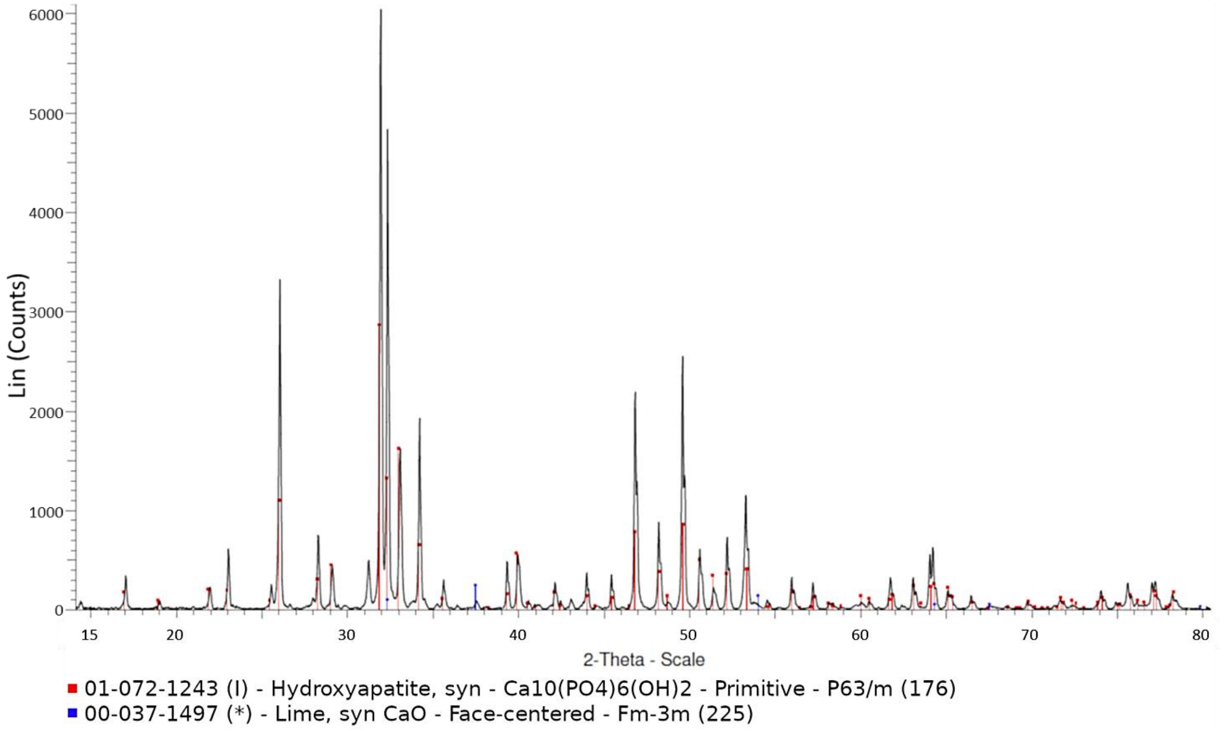

Supplementary Figure 1. X-ray diffraction pattern for hydroxyapatite pellet produced by the method of Ooi, C. et al. [1]. The hydroxyapatite structure was confirmed in the sample as a small amount of CaO. There was no evidence of hydroxyapatite degradation (β-TCB) at high temperatures.

References

[1] C. Y. Ooi, M. Hamdi, and S. Ramesh, *Properties of hydroxyapatite produced by annealing of bovine bone,* Ceram. Int., vol. 33, no. 7, pp. 1171–1177, Sep. 2007.
